# The Level of Residual Dispersion Variation and the Power of Differential Expression Tests for RNA-Seq Data

**DOI:** 10.1371/journal.pone.0120117

**Published:** 2015-04-07

**Authors:** Gu Mi, Yanming Di

**Affiliations:** 1 Department of Statistics, Oregon State University, Corvallis, Oregon, United States of America; 2 Molecular and Cellular Biology Program, Oregon State University, Corvallis, Oregon, United States of America; University of Manchester, UNITED KINGDOM

## Abstract

RNA-Sequencing (RNA-Seq) has been widely adopted for quantifying gene expression changes in comparative transcriptome analysis. For detecting differentially expressed genes, a variety of statistical methods based on the negative binomial (NB) distribution have been proposed. These methods differ in the ways they handle the NB nuisance parameters (i.e., the dispersion parameters associated with each gene) to save power, such as by using a dispersion model to exploit an apparent relationship between the dispersion parameter and the NB mean. Presumably, dispersion models with fewer parameters will result in greater power if the models are correct, but will produce misleading conclusions if not. This paper investigates this power and robustness trade-off by assessing rates of identifying true differential expression using the various methods under realistic assumptions about NB dispersion parameters. Our results indicate that the relative performances of the different methods are closely related to the level of dispersion variation unexplained by the dispersion model. We propose a simple statistic to quantify the level of residual dispersion variation from a fitted dispersion model and show that the magnitude of this statistic gives hints about whether and how much we can gain statistical power by a dispersion-modeling approach.

## Introduction

Over the last ten years, RNA-Sequencing (RNA-Seq) has become the technology of choice for quantifying gene expression changes in comparative transcriptome analysis [1]. The negative binomial (NB) distribution has been widely used for modeling RNA-Seq read counts [2–4]. Although early studies have shown that the Poisson model is adequate for modeling RNA-Seq count variation from *technical* replicates [5], many recent RNA-Seq analyses revealed that RNA-Seq counts from *biological* replicates show significant extra-Poisson variation. The NB distribution can be derived as a mixture of Poisson distributions in the so-called Gamma-Poisson model. For a random variable *Y* having an NB distribution with mean *μ* and dispersion *ϕ*, the variance is given by Var(*Y*) = *μ* + *ϕμ* ^2^, and the dispersion parameter *ϕ* determines the extent to which the variance exceeds the mean. The square root of *ϕ* is also termed “biological coefficient of variation” (BCV) in [6].

The dispersion *ϕ* is a nuisance parameter in tests for differential expression (DE), but correct estimation of *ϕ* is essential for valid statistical inference. In a typical RNA-Seq experiment, our ability to detect truly DE genes is hampered by the large number of genes, the small sample size, and the need to estimate the dispersion parameters. To ameliorate this difficulty, many different NB dispersion models have been proposed (see the Background section for more details) with a common theme of “pooling information across genes”. An NB dispersion model relates the dispersion to some measure of read abundance, *a*, through a simple parametric or smooth function *f* with a small number of parameters *α* (estimated from data):

$$\begin{array}{c} \log\left(\phi_{ij}\right)=f\left(a_{ij};\alpha \right), \end{array}$$ (1)

where *i* indexes genes and *j* indexes biological samples. For example, in [4] we let *a* be preliminarily estimated mean relative frequencies and let *f* be a linear or quadratic function of log(*a*). This and other dispersion models are motivated by empirical evidence of a trend—over all genes—of decreasing size of dispersion parameter with increasing relative frequency of RNA-Seq reads for the genes. By introducing a dispersion model *f*, one hopes to summarize the dispersion parameters for all genes by a small number of model parameters *α* and thus drastically reduce the number of nuisance parameters to estimate. A dispersion-modeling approach as described above can lead to power saving, *if* a correct or “close enough” model is used. While empirical evidence overwhelmingly suggests a general trend between dispersion level and mean expression, goodness-of-fit measures [6, 7] suggest simple parametric and smooth function models may not be able to capture the total variation in dispersion (see the subsection “Background/Goodness-of-Fit Tests” for more details).

The key question that motivates this study is, even when a dispersion model shows lack-of-fit, to what degree can it still be useful in improving the power of the DE test. It will be convenient for us to consider a general trend in dispersion parameter, but also allow for variation about the trend, as follows:

$$\begin{array}{c} \log\left(\phi_{ij}\right)=f\left(a_{ij};\alpha \right)+\epsilon_{i}, \end{array}$$ (2)

where *ε* represents an individual component in *ϕ* that is unexplained by the trend. Intuitively, the strategy of “pooling information across genes” through a dispersion model *f* will be most effective if the overall level of residual variation in *ε* is low. In this paper, as an approximation, we model *ε* using a normal distribution *ε*_*i*_ ∼ 𝒩(0,*σ* ^2^) and quantify the level of variation in *ε* by *σ* ^2^. We estimate *σ* for five real RNA-Seq datasets (from human, mouse, zebrafish, Arabidopsis and fruit fly) and then investigate the power and robustness of DE tests when the amount of residual variation in dispersion matches that from the real data. We also explore how the relative performances of different DE test methods will change as the magnitude of *σ* changes.

In this paper, we focus on the overall level of deviation (summarized by *σ*) from an estimated model for log dispersion. Zhou *et al.*[8] discussed the impact of “outliers”—a small number of highly influential outlining cases—on the performance of DE test. Under our framework, it is possible to investigate the impact of such individual outliers by considering non-normal models (such as a binomial or Poisson point process model) for *ε*, but such extensions are nontrivial and we will not pursue them in this paper. Our approach for estimating *σ* ^2^ is related to the empirical Bayes approach for estimating *ε* under a normal prior distribution. However, our focus in this paper is in estimating *σ* ^2^, not the individual $\varepsilon_{i}^{\prime }s$. The quantity *σ* ^2^ is related to the quantity *d*_0_ discussed in [9]. We explain this connection in more details in the subsection “Background/Weighted Likelihood and Empirical Bayes Methods”.

## Background

### RNA-Seq

In brief, a typical RNA-Seq pipeline can be summarized as follows: purified RNA samples are converted to a library of cDNA with attached adaptors, and then sequenced on an HTS platform to produce millions of short sequences from one or both ends of the cDNA fragments. These reads are aligned to either a reference genome or transcriptome (called sequence mapping), or assembled *de novo* without the genomic sequence. The aligned reads are then summarized by counting the number of reads mapped to the genomic features of interest (e.g., exons or genes), and the expression profile is eventually represented by a matrix of read counts (non-negative integers) where rows are genes (or some other genomic features like exons) and columns are samples. Subsequent steps that rely heavily on statistical analyses include normalization of reads and testing DE genes between samples under different environmental or experimental conditions.

### NB Regression Models

An NB regression model for describing the mean expression as a function of explanatory variables includes the following two components:

1. An NB distribution for the individual RNA-Seq read counts *Y*_*ij*_:
  $$\begin{array}{c} Y_{ij}∼\text{NB}\left(\mu_{ij},\phi_{ij}\right), \end{array}$$
  where *i* = 1,…,*m* indexes genes, *j* = 1,…,*n* indexes samples, *μ*_*ij*_ is the mean, and *ϕ*_*ij*_ is the dispersion parameter such that $\text{Var}(Y_{ij})=\mu_{ij}+\phi_{ij}\mu_{ij}^{2}$.
2. A log-linear regression model for the mean *μ*_*ij*_ as a function of *p* explanatory variables *X*_*jk*_ (*k* = 1,…,*p*):
  $$\begin{array}{c} \log\left(\mu_{ij}\right)=\log\left(N_{j}\right)+\log\left(R_{j}\right)+\sum_{k=1}^{p}\beta_{ik}X_{jk}. \end{array}$$ (3)

These two components resemble a generalized linear model (GLM) [10], but note that the dispersion *ϕ*_*ij*_ is unknown (see the “NB Dispersion Models” subsection below). The two additive constants, log(*N*_*j*_) and log(*R*_*j*_), have to do with count normalization: accounting for different observed library sizes (*N*_*j*_) and the apparent reduction/increase in the expression levels of non-DE genes resulting from the increased/decreased expression of a few truly DE genes [3, 11]. The normalization constants, *N*_*j*_ and *R*_*j*_, are pre-estimated and treated as known during GLM fitting. In many applications, the same constant (*N*_*j*_ *R*_*j*_) is assumed for all genes in a sample, but it may be advantageous to introduce between-gene normalization factors to account for some gene-specific sources of technical biases such as GC-content and gene length [12]. Between-gene normalization can be incorporated into the GLM framework as well. See [13–15] for relevant discussions.

### DE Tests

Testing differential expression can often be reduced to testing that one or more of the regression coefficients equal zero. For example, for comparing gene expression levels between two groups, we can let *p* = 2, *X*_*j*1_ = 1 for all *j*; *X*_*j*2_ = 1 if sample *j* is from group 2 and *X*_*j*2_ = 0 if sample *j* is from group 1. Under this parameterization, *β*_1_ corresponds to group 1’s relative mean expression level and *β*_2_ corresponds to the log fold change between group 2 and group 1. The null hypothesis is *H*_0_:*β*_2_ = 0.

In general NB regression settings, exact tests are not available, but asymptotic tests, such as likelihood ratio test, can be used. Di *et al.* [16, 17] showed that the performance of likelihood ratio test in small sample settings can be improved with higher-order asymptotics (HOA) adjustment. Lund *et al.* [18] discussed quasi-likelihood (QL) methods by replacing likelihood ratio test with QL *F*-test for better FDR control, where the test statistic is based on quasi-dispersion parameter estimates or two variants called QLShrink and QLSpline for pooling information across genes.

### NB Dispersion Models

As mentioned in the Introduction section, many current DE analysis methods use an NB dispersion model to capture the general trend between dispersion and read abundance. The different DE analysis methods can be put into the following general categories according to the functional form *f* of the dispersion model and the treatment of individual variation (see Equation (2)):

1. Common: Earlier works of Robinson and Smyth [19] discussed a common dispersion model where *f* is a constant. In other words, *ϕ*_*ij*_ = *c* for all *i*, *j*.
2. Parametric function: Recognizing an evident trend between the dispersion and relative gene expression, Di *et al.* [4] adopted a parametric NBP model where the log dispersions are modeled as a linear function of the log relative mean frequencies. Referring to Equation (1), in an NBP model, $a_{ij}=\pi_{ij}=\frac{\mu_{ij}}{N_{j}R_{j}}$ and *f*(*a*_*ij*_;*α*) = *α*_0_ + *α*_1_log(*π*_*ij*_). A natural extension to NBP is the NBQ model which incorporates an extra quadratic term:
  $$\begin{array}{c} f\left(a_{ij};\alpha \right)=\alpha_{0}+\alpha_{1}\log\left(\pi_{ij}\right)+\alpha_{2}{\left[\log(\pi_{ij})\right]}^{2}. \end{array}$$ (4)
3. Smooth function: Anders and Huber [3] suggested fitting a non-parametric curve to capture the dispersion-mean dependence. McCarthy *et al.* [6] introduced a similar “trended” (non-parametric) model. NBPSeq added an NBS model for non-parametric smooth dispersion model.

The methods above ignore possible individual dispersion variation (i.e., *ε*_*i*_ in Equation (2)) in subsequent DE tests.

4 Shrinkage methods: McCarthy *et al.* [6] discussed options to use weighted average between genewise dispersion estimates and trended estimates in an empirical Bayes framework (we will call this method “tagwise-trend”). The genewise estimates can also be shrunk towards a common value [20]. Love *et al.* [12] added a shrinkage option in DESeq2.
5 Quasi-likelihood methods: Lund *et al.* [18] suggested fitting a quasi-likelihood (QL) model by specifying (for gene *i* and sample *j*):
  $$\begin{array}{c} \mathrm{Var}\left(Y_{ij}\right)=\Phi_{i}V_{i}\left(\mu_{ij}\right), \end{array}$$ (5)
  with the NB variance function $V_{i}(\mu_{ij})=\mu_{ij}+\omega_{i}\mu_{ij}^{2}$. Both the NB dispersion parameter (*ω*_*i*_) and the quasi-likelihood dispersion parameter (Φ_*i*_) are estimated from the data and used to model the variance of the read count *Y*_*ij*_. The QL-dispersion Φ_*i*_ adjusts for degrees of freedom and accounts for uncertainty in the estimated NB variance. A shrinkage method is used to estimate Φ_*i*_ and two variants, “QLShrink” and “QLSpline”, differ in the formulation of prior distribution of Φ_*i*_. These QL-based approaches are implemented in the QuasiSeq package. (See also, the review in the subsection “Weighted Likelihood and Empirical Bayes Methods” below.)
6 Genewise: The NBPSeq package allows for fitting NB regression model and performing DE test to each gene separately without assuming any dispersion model. HOA adjustment is used to improve the performance of the likelihood ratio test.

In the above, we mainly summarized methods implemented in the R/Bioconductor packages DESeq, DESeq2, edgeR, NBPSeq and QuasiSeq[21, 22]. They represent the wide range of currently available options. These packages use slightly different predictors (*a*_*ij*_ in Equation (1)) in their dispersion models, and also use different methods to estimate dispersion models, but these differences are of no primary interest in our power-robustness analysis. As we will see later, the main factor that influences the DE test performance is how the individual dispersion variation is handled.

### Goodness-of-Fit Tests

Mi *et al.* [7] discussed a resimulation-based goodness-of-fit (GOF) test for negative binomial models fitted to individual genes, and then extended the test to multiple genes using Fisher’s method for combining *p*-values. The paper also introduced diagnostic plots for judging GOF. McCarthy *et al.* [6] transformed genewise deviance statistics to normality and used QQ-plot to examine GOF of different dispersion models. In particular, their QQ-plots (Fig. 2 in their paper) indicated that simple dispersion models, such as a common or trended dispersion model, showed lack-of-fit when used to model an RNA-Seq dataset from a study on oral squamous cell carcinomas (OSCC). One question that motivated this study is how different DE test methods perform when the fitted dispersion model (the trend part) shows lack-of-fit. Intuitively, the performance of different test methods, especially the ones that do not explicitly account for individual residual variation, should be related to the level of residual dispersion variation. We want to make this statement more precise. This motivated us to quantify the level of residual dispersion variation using *σ* ^2^ and relate the power/robustness analysis to the magnitude of *σ* ^2^.

### Weighted Likelihood and Empirical Bayes Methods

In the edgeR package, one can estimate the genewise (or tagwise) dispersion by maximizing the weighted average of two adjusted profile likelihoods:

$$\begin{array}{c} {\mathrm{APL}}_{i}\left(\phi_{i}\right)+G_{0}\cdot {\mathrm{APL}}_{S}\left(\phi_{i}\right), \end{array}$$ (6)

where APL_*i*_ is computed from each gene separately, and APL_*S*_ represents the general trend in mean-dispersion dependence. The detailed formulation of APL_*S*_(*ϕ*_*i*_) has been evolving over the years. For example, it can be formed by a (weighted) average of APL_*i*_ values for genes near *i*. This weighted likelihood method has its root in empirical Bayes method and APL_*S*_ serves as the prior likelihood [6, 9, 20].

To estimate *G*_0_, Chen *et al.* [9] considered an empirical Bayes approach using quasi-likelihood. A variance function *V*(*μ*) was used to specify the mean-variance relationship according to, for example, a Poisson or a negative binomial model, and a quasi-likelihood function:

$$\begin{array}{c} \mathrm{Var}\left(Y_{ij}\right)=\sigma_{i}^{2}\cdot V\left(\mu_{ij}\right) \end{array}$$ (7)

was used to model the additional variation in the mean-variance relationship between genes (they indexed genes with letter *g* while we use *i* in this paper). Chen *et al.* [9] assumed a scaled inverse *χ* ^2^ prior distribution of $\sigma_{i}^{2}$:

$$\begin{array}{c} \sigma_{i}^{2}∼s_{0}^{2}\cdot \frac{d_{0}}{\chi_{d_{0}}^{2}} \end{array}$$

with parameters $s_{0}^{2}$ and *d*_0_. In comparison, the model (Equation (2)) in this paper is on the dispersion parameter. The parameter *d*_0_ is called the *prior degrees of freedom* and it plays an analogous role as *σ* ^2^ in this paper. For a series of simulated datasets, our estimates of *σ* ^2^ is approximately inversely proportional to estimates of *d*_0_ as explained below (see Fig. E in the Supporting Information S1 File).

Under an empirical Bayes framework, the parameters of the prior distribution are estimated from the data. Let *D*_*i*_ be the residual deviance of the generalized linear model fitted to read counts and *d*_*i*_ be the known effective residual degrees of freedom for gene *i*. Chen *et al.* [9] explained that given $\sigma_{i}^{2}$, the mean residual deviance, defined as

$$\begin{array}{c} s_{i}^{2}=\frac{1}{d_{i}}D_{i}, \end{array}$$

has, approximately, a scaled chi-square conditional distribution:

$$\begin{array}{c} s_{i}^{2}|\sigma_{i}^{2}∼\sigma_{i}^{2}\frac{\chi_{d_{i}}^{2}}{d_{i}}. \end{array}$$

It then follows that the marginal distribution of $s_{i}^{2}$ is a scaled *F*-distribution:

$$\begin{array}{c} s_{i}^{2}∼s_{0}^{2}\cdot F_{d_{i},d_{0}}. \end{array}$$

$s_{0}^{2}$ and *d*_0_ can be estimated from $s_{i}^{2}$ using the method of moments. Chen *et al.* [9] suggested that one can use $\frac{d_{0}}{d_{i}}$ as *G*_0_ in the weighted likelihood (Equation (6)). Recent versions of edgeR provide this option. However, for the simulations performed in this paper, when performing DE tests using edgeR, we estimated the dispersion parameters using the edgeR functions estimateGLMTrendedDisp and estimateGLMTagwiseDisp, where similar weighted likelihood was considered, but the default value *G*_0_ = 10 was used (see also McCarthy *et al.* [6]).

The variance function (*V*(*μ*)) and quasi-likelihood function (7) described above are essentially the same ones as considered in [18] (cf. Equation (5)), but the estimation methods and the definition of *d*_*i*_ used in the two papers were slightly different (e.g., one of the reviewers pointed out that a refinement was made in Chen *et al.* [9] where *d*_*i*_ is decreased slightly to allow for bias in the residual deviance associated with exact zero counts). In [18], the estimated *d*_0_ was used for constructing the quasi-likelihood *F*-test. Wu *et al.* [23] proposed another empirical Bayes shrinkage estimator for the dispersion parameter which aimed to adequately capture the heterogeneity in dispersion among genes. The empirical Bayes strategy has also been used in [24] for modeling microarray data.

### Other Related Work

There are also recent works on comparing the performances of DE tests: Soneson and Delorenzi [25] evaluated 11 tools for their ability to rank truly DE genes ahead of non-DE genes, the Type-I error rate and false discovery rate (FDR) controls, and computational times. Landau and Liu [26] discussed dispersion estimation and its impact on DE test performance, mainly focusing on different shrinkage strategies (none, common, tagwise or maximum). The key aspects of this paper are to explicitly quantify the level of inadequacy of a fitted dispersion model using a simple statistic, and to link the magnitude of this statistic directly to the performance of the associated DE test.

## Results

We investigate the power and robustness of DE tests under realistic assumptions about the NB dispersion parameters. We fit the NBQ dispersion model (see Equation (4)) to real datasets to capture the general trend in the dispersion-mean dependence. We model the residual variation in dispersion using a normal distribution (see Equation (2)) and the level of residual variation is then summarized by a simple quantity, the normal variance *σ* ^2^. Because biological variations are likely to differ across species, and experiments involve varied sources of uncertainty, we choose to analyze five datasets from different species that represent a broad range of characteristics and diversity for typical RNA-Seq experiments. The species include human (*Homo sapiens*), mouse (*Mus musculus*), zebrafish (*Danio rerio*), Arabidopsis (*Arabidopsis thaliana*) and fruit fly (*Drosophila melanogaster*). The Methods section includes descriptions of the datasets. For each experiment/dataset, unless otherwise specified we will provide the following results:

1. Mean-dispersion plot with trends estimated from NB dispersion models;
2. Gamma log-linear regression as informal model checking;
3. Estimation of the variance *σ* ^2^ of dispersion residuals from a fitted dispersion model;
4. Power-robustness evaluations of DE tests using datasets simulated to mimic real datasets.

The main focus of this paper is on the quantification of the level of residual dispersion variation and power-robustness investigation under realistic settings (3 and 4 above). The diagnostic plots and statistics (1 and 2 above) are useful in routine analysis of RNA-Seq data, and they also help us verify that the NBQ dispersion model largely captures the general trend in the dispersion-mean dependence.

Anders *et al.* [27] suggested removing genes with less than or equal to one read per million (rpm) in at least *n* of the samples, where *n* is the size of the smallest group of replicates. We follow a similar criterion but set *n* = 1 in order to keep more (lowly-expressed) genes in study. In R, this is achieved by subsetting the row indices by rowSums(cpm(data)>1)>=1. The library size adjustments are computed for genes passing this criterion.

### Mean-Dispersion Plots with Estimated Trends from Dispersion Models

**Fig 1:**
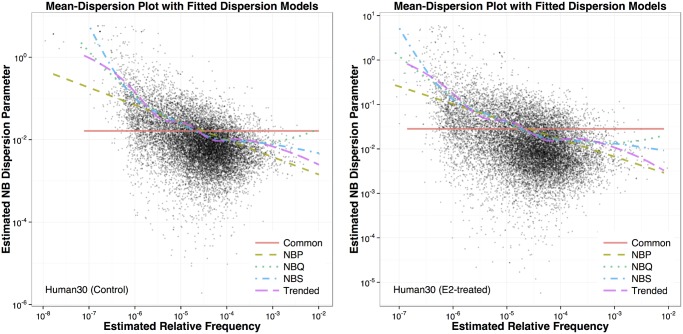
Mean-dispersion plots for the human RNA-Seq dataset. The left panel is for the control group and the right panel is for the E2-treated group. Each group has seven biological replicates. The sequencing depth for this dataset is 30 million. Each point on the plots represents one gene with its method-of-moment (MOM) dispersion estimate (${\hat{\phi }}^{\text{MOM}}$) on the *y*-axis and estimated relative mean frequency on the *x*-axis. The fitted curves for five dispersion models are superimposed on the scatter plot.

Fig. 1 shows the mean-dispersion plots for the two treatment groups in the human dataset (with sequencing depth of 30 million). In each plot, method-of-moment (MOM) estimates (${\hat{\phi }}^{\text{MOM}}$) of the dispersion *ϕ* for each gene are plotted against estimated relative mean frequencies (on the log-log scales). For each gene *i*, ${\hat{\phi }}_{i}^{\text{MOM}}$ is defined as $\overset{n}{\underset{j=1}{\sum }}\frac{\left[{\left(y_{ij}-{\tilde{\mu }}_{i}\right)}^{2}-{\tilde{\mu }}_{i}\right]}{n{\tilde{\mu }}_{i}^{2}}$, where *y*_*ij*_ are the read counts and ${\tilde{\mu }}_{i}$ is their mean. Note that for this dataset, the library sizes (column totals) are roughly the same. Genes with ${\hat{\phi }}_{i}^{\text{MOM}}\leq 0$ were not used in the mean-dispersion plots and the gamma log-linear regression analysis. We also overlaid the trends from five fitted dispersion models representing the wide range of currently available options: common, NBP, NBQ, NBS and trended (see the “Background/NB Dispersion Models” subsection above). We make the following remarks:

#1 The fitted NBP, NBQ, NBS and trended dispersion models all capture the overall decreasing trend in the MOM genewise estimates.
#2 The fitted models agree more in the mid-section of the expression distribution and less in the tails where genes have extremely low or high expression levels. This kind of behavior is common in non-parametric smooth estimates and regression models, and it has some implications on how we design the power simulations later.
#3 Such mean-dispersion plots are informative in checking how different dispersion models may potentially over-/under-estimate the dispersion parameters, which in turn will influence DE test results.
#4 Note that the deviation of the genewise MOM estimates from the fitted dispersion models is *not* the same as the *ε* in Equation (2), since this deviation also reflects the additional estimation error due to small sample size.

Mean-dispersion plots for the other four datasets show similar features and are included in Figs. A–D of the Supporting Information S1 File.

### Gamma Log-Linear Regression Analysis

As informal model checking, we fit polynomial gamma log-linear regression models of ${\hat{\phi }}^{\text{MOM}}$ on $\log(\hat{\pi })$. Table 1 summarizes the variability in the logged genewise dispersion estimates $\log({\hat{\phi }}^{\text{MOM}})$ explained by the linear, quadratic and cubic models (results shown for the control group only and without pre-filtering lowly-expressed genes). The proportion of variation in $\log({\hat{\phi }}^{\text{MOM}})$ explained by the fitted models varies across species (e.g., for the quadratic fit, it ranges from 31% to 75%) and also depends on sequencing depths. The quadratic regression model improves over the simple linear regression model by explaining an additional 2% to 11% of variation, while adding a cubic term has almost negligible effects.

**Table 1:** Proportion of variation in $\log({\hat{\phi }}^{\text{MOM}})$ explained by fitted models.

	**Dataset**					
**Model**	Human5	Human30	Mouse	Zebrafish	Arabidopsis	Fruit Fly
Linear	73.09%	72.29%	49.20%	32.08%	36.30%	23.79%
Quadratic	75.15%	74.38%	54.85%	43.02%	41.02%	31.20%
Cubic	75.46%	74.45%	54.55%	43.77%	41.01%	32.74%

The proportion of variation in $\log({\hat{\phi }}^{\text{MOM}})$ explained by the fitted gamma log-linear, quadratic and cubic regression models. Results are shown for the control group only.

### Quantification of the Level of Residual Dispersion Variation

As discussed in the Introduction section, we model the dispersion residuals using a normal distribution, $\varepsilon =\log(\phi )-\log(\hat{\phi })∼𝒩(0,\sigma^{2})$, and thus quantify the level of residual variation using *σ* ^2^ or equivalently *σ*. Using the approach described in the Methods section, we estimate *σ* from each of the five real datasets after fitting an NBQ dispersion model (see Equation (4)). Table 2 summarizes the estimates and the corresponding standard errors. The magnitudes of $\hat{\sigma }$ indicate that the fitted dispersion models do not fully explain the total variation in the dispersion. The NBQ dispersion model uses estimated mean relative frequencies (${\hat{\pi }}_{ij}$) as predictors, and the results here suggest that there is still substantial individual variation among genes with the same values of ${\hat{\pi }}_{ij}$.

**Table 2:** Estimated level of residual dispersion variation in five real RNA-Seq datasets.

**Dataset**	**#samples**	**MLE $\hat{\sigma }$**	**SE$\left(\hat{\sigma }\right)$**
Human30	(7, 7)	1.021	0.014
Mouse	(3, 3)	1.228	0.022
Zebrafish	(4, 4)	1.105	0.020
Arabidopsis	(3, 3)	0.956	0.021
Fruit fly	(4, 3)	1.015	0.016

The columns are: name of the dataset, the number of samples (control, treatment), the maximum likelihood estimate (MLE) $\hat{\sigma }$, and the standard error (SE) of $\hat{\sigma }$.

It is possible to turn the estimate $\hat{\sigma }$ into a goodness-of-fit test for the fitted dispersion model. However, we want to ask whether a dispersion model is useful even when the fitted model shows lack-of-fit. For this purpose, the quantitative measure $\hat{\sigma }$ is more intuitive than a test *p*-value, since it directly reflects the degree of deviation from the fitted dispersion model. In the next section, we will explore the connection between the magnitude of $\hat{\sigma }$ and the performance of DE tests in terms of power and FDR.

### Power-Robustness Evaluations

We compare the power and FDR/Type-I error control of a range of DE test methods on datasets simulated to mimic the five real datasets.

#### Simulation Setup

In our power-robustness analysis, we will compare performance of six DE test methods. We choose one representative method from each of the categories summarized in the “Background/NB Dispersion Models” subsection (prefixed with the name of the R/Biconductor package that implements the method, and a colon): NBPSeq:genewise, edgeR:common, NBPSeq:NBQ, edgeR:trended, edgeR:tagwise-trend, and QuasiSeq:QLSpline. These methods represent a range of available options on how to handle the dispersion estimation. The edgeR:common method is included solely for benchmark purpose as it is over-simplified and not recommended for practical use. The NBPSeq:NBQ method represents parametric dispersion models and the NBQ dispersion model generally provides better fit than the simpler NBP model [7]. The edgeR:tagwise-trend method represents the empirical Bayes shrinkage methods [6]. The QuasiSeq:QLSpline method represents quasi-likelihood methods [18]. These methods also use different tests for DE analysis. For testing DE, methods from edgeR use likelihood ratio test, methods from NBPSeq use likelihood ratio test with HOA adjustment, and the QuasiSeq:QLSpline method uses QL *F*-test. Table 3 provides a summary of the DE test methods compared.

**Table 3:** Summary of DE test methods compared.

**DE Test Method**	**Trend *f***	**Consider *ε*?**	**Test**	**Note**
NBPSeq:Genewise	n/a	yes	LRT	HOA adjustment
edgeR:Common	constant	no	LRT	n/a
NBPSeq:NBQ	parametric	no	LRT	HOA adjustment
edgeR:Trended	smooth	no	LRT	n/a
edgeR:Tagwise-trend	smooth	yes	LRT	empirical Bayes shrinkage
QuasiSeq:QLSpline	smooth	yes	QL	degrees-of-freedom adjustment

The columns are: name of the DE test method, the functional form *f* used for capturing the general trend in dispersion, whether the method considers individual dispersion variation unexplained by the trend, the test statistic used (LRT: likelihood ratio test; QL: quasi-likelihood method), and additional notes about the test (n/a if not applicable).

We simulate two-group comparison datasets that mimic the five real RNA-Seq datasets. From each real dataset, we randomly select 5,000 genes and fit NB regression models to them (see Equation (3) and the “Background/DE Tests” subsection above). We generate a new dataset of 5,000 genes based on fitted models. We specify the mean expression levels based on estimated ${\hat{\beta }}_{ik}$, with *R*_*j*_ = 1 and *N*_*j*_ reflecting the sequencing depth (e.g., *N*_*j*_ = 2.5×10^7^ for the human dataset and 1.5×10^7^ for the mouse dataset). For all genes, we set *β*_*i*1_ as the estimated value from the real data. If gene *i* is designated as DE, we either use ${\hat{\beta }}_{i2}$ estimated from the real data as its log fold change (i.e., we set $\beta_{i2}={\hat{\beta }}_{i2}$), or let *β*_*i*2_ correspond to fixed fold changes of 1.2 or 1.5. For any non-DE gene *i* ^′^, we set *β*_*i*^′^2_ = 0. In real data analysis, it is unknown which genes are DE. For each dataset, we randomly designate *m*_1_ genes as DE. We consider two levels, 0.1 and 0.2, for the percentage of DE genes (*π*_1_ = *m*_1_/*m*). Approximately (when using estimated DE fold changes) or exactly (when using fixed DE fold changes) half of the simulated DE genes are over-expressed and half are under-expressed. Early microarray studies had shown that a smaller proportion of DE genes tend to make it more difficult to control FDR at the nominal level [28].

We specify the dispersion parameters according to Equation (2) with the trend part, *f*(*a*_*ij*_;*α*), being the fitted NBQ model (fitting Equation (4) to real data). The deviation from the trend is controlled by *ε*_*i*_ and will be simulated according to a 𝒩(0,*σ* ^2^) distribution. We want to choose *σ* ^2^ to match the real data, but there is some subtlety in how to achieve this: in practice, when fitting the NBQ model, we use the fitted values ${\hat{\pi }}_{ij}$ as the predictors since true *π*_*ij*_ values are not available, but when we simulate counts, the ${\hat{\pi }}_{ij}$ values are not available. Our solution is to use *π*_*ij*_ as predictor in the NBQ model when simulating *ε*, but choose $\sigma =\tilde{\sigma }$ through a *calibration* approach such that if we were to fit the NBQ model to the simulated data later—using the estimated ${\hat{\pi }}_{ij}$ as predictor, the estimated $\hat{\sigma }$ would match the one estimated from the real data (also using the estimated ${\hat{\pi }}_{ij}$ as predictor). The estimated values of $\hat{\sigma }$ from real datasets are summarized in Table 2. The calibrated values $\tilde{\sigma }$ and the details about the calibration approach are presented in the Methods section. In our simulations, we will consider different levels of residual dispersion variation and set *σ* to $\tilde{\sigma }$, $0.5\tilde{\sigma }$ or 0.

There are other factors that may potentially contribute to the difference in DE test performance, such as the presence of outliers, the proportion of up and down-regulated genes, potential correlation between gene expression levels, to just name a few. In this paper, we will focus on the impact of unmodeled dispersion variation on DE test performance.

#### Power Evaluation

For power evaluation, we plot true positive rates (TPR) versus false discovery rates (FDR). For a DE test, a true positive (TP) indicates the test correctly identifies a DE gene; a false positive (FP) indicates the test incorrectly identifies a non-DE gene as DE; and a false negative (FN) indicates the test incorrectly declares a DE gene as non-DE. The TPR and FDR are defined as: TPR = TP/(TP + FN) and FDR = FP/(TP + FP). A TPR-FDR curve contains equivalent information as a precision-recall curve or an ROC curve, but focuses on the relationship between TPR (power) and FDR. The power of a DE test depends on the alternative hypothesis and will likely vary between genes. The TPR reflects the average power of a test to detect truly DE genes in a simulated dataset. If we compare the TPR of the tests at the same FDR level, we are essentially comparing the size-corrected power.

**Fig 2:**
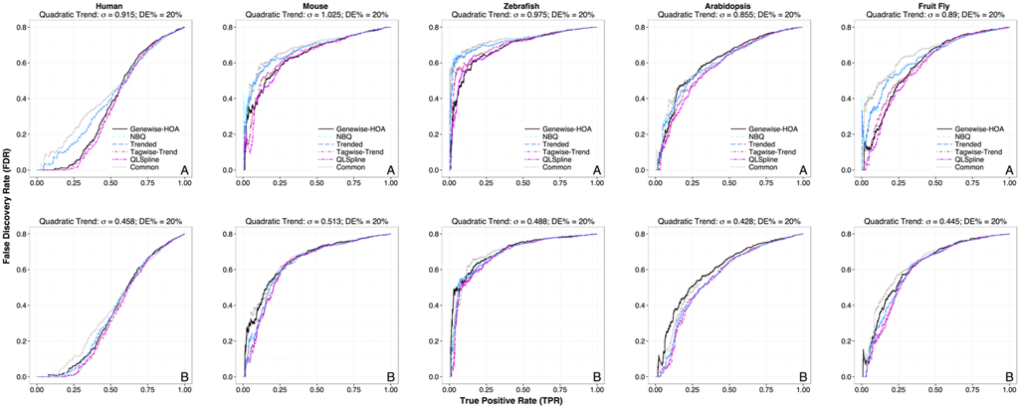
True Positive Rate (TPR) vs. False Discovery Rate (FDR) plots for the six DE test methods performed on RNA-Seq datasets simulated to mimic real datasets. The fold changes of DE genes are estimated from real data. The columns correspond to the following datasets (left to right) used as templates in the simulation: human, mouse, zebrafish, Arabidopsis, and fruit fly. The level of residual dispersion variation, *σ*, is specified at the estimated value ($\tilde{\sigma }$) in panels labeled with A (first row), and half the estimated value ($0.5\tilde{\sigma }$) in panels labeled with B (second row). In each plot, the *x*-axis is the TPR (which is the same as recall and sensitivity) and the *y*-axis is the FDR (which is the same as one minus precision). The percentage of truly DE genes is specified at 20% in all datasets. The FDR values are highly variable when TPR is close to 0, since the denominator TP + FP is close to 0.

The upper row of Fig. 2 shows the TPR-FDR plots for the six tests performed on each of the five datasets simulated to mimic the five real datasets. In particular, the simulated datasets have the same level of residual dispersion variation *σ* ^2^ as estimated from the five real datasets, and the fold changes of DE genes are also estimated from real data. A better method will have its TPR-FDR curve closer to the lower-right corner, indicating a lower FDR for achieving a fixed power, or a higher power for a fixed tolerable FDR. For four of the datasets, the QuasiSeq:QLSpline, edgeR:tagwise-trend and NBPSeq:genewise methods outperform the NBPSeq:NBQ, edgeR:trended and edgeR:common methods, with the edgeR:common method being the worst. For the simulation dataset based on the Arabidopsis real dataset, no test dominates at all FDR levels.

It is somewhat surprising that the performance of the simple NBPSeq:genewise method is comparable to the best methods in all cases. This indicates that if the level of residual dispersion variation is as high as the estimated (see Table 2), the potential power saving through dispersion modeling is quite limited.

The relative performance of the tests will change if the level of residual dispersion variation (*σ* ^2^) changes. The lower row of Fig. 2 shows the TPR-FDR plots when *σ* is simulated to be half the estimated values ($\sigma =0.5\tilde{\sigma }$), again with DE fold changes estimated from real data. The performance of the NBPSeq:NBQ and trended methods has much improved and is better than the NBPSeq:genewise method in three of the datasets (the ones based on mouse, zebrafish and Arabidopsis). When we further reduced *σ* to 0 in our simulations, all methods outperformed the NBPSeq:genewise approach. The QuasiSeq:QLSpline and edgeR:tagwise-trend methods managed to perform consistently well as we vary the magnitude of *σ*.

**Fig 3:**
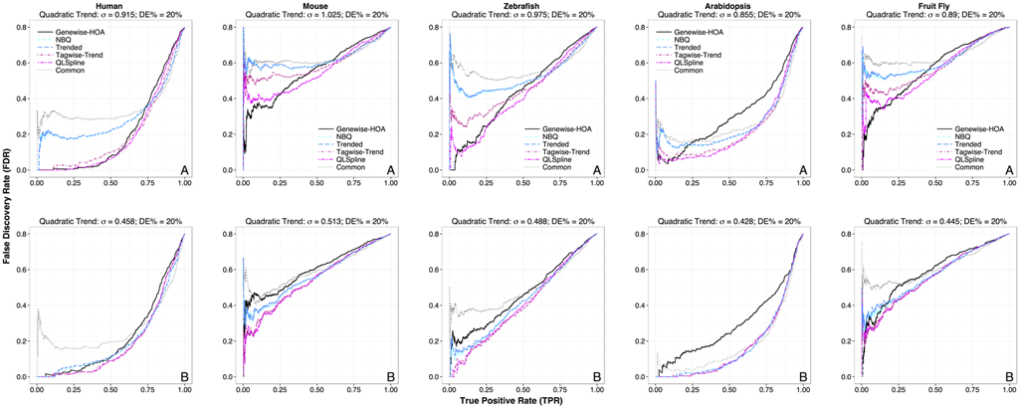
True Positive Rate (TPR) vs. False Discovery Rate (FDR) plots for the six DE test methods performed on RNA-Seq datasets simulated to mimic real datasets. The fold changes of DE genes are fixed at 1.2 (half of the DE genes are over-expressed and the other half are under-expressed). Other simulation settings are identical to those described in Fig. 2 legend.

**Fig 4:**
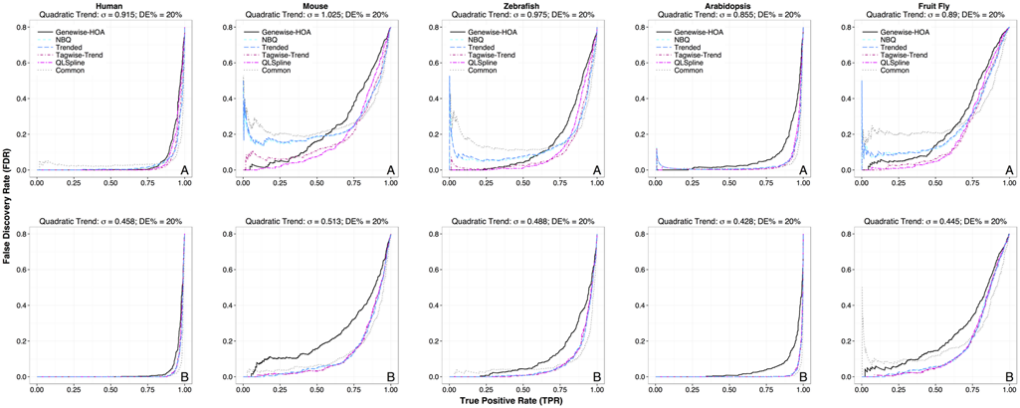
True Positive Rate (TPR) vs. False Discovery Rate (FDR) plots for the six DE test methods performed on RNA-Seq datasets simulated to mimic real datasets. The fold changes of DE genes are fixed at 1.5 (half of the DE genes are over-expressed and the other half are under-expressed). Other simulation settings are identical to those described in Fig. 2 legend.

To understand how each method performs under a wide range of situations, we also performed simulations where the fold changes for DE genes were fixed instead of estimated from real data, while other settings (e.g., the percentage of DE genes, *σ* and $\tilde{\sigma }$) remained the same as before. Figs. 3 and 4 show the TPR-FDR plots when the fold changes of DE genes were fixed at 1.2 (low) and 1.5 (moderate) respectively. In general, the NBPSeq:genewise, edgeR:tagwise-trend and QuasiSeq:QLSpline perform better than edgeR:common, NBPSeq:NBQ and edgeR:trend, which is consistent with the observations when the fold changes are estimated from real data. In the low DE fold change case and when the residual dispersion variation is as estimated (upper row of Fig. 3), there is more separation between the QuasiSeq:QLSpline method and the edgeR:tagwise-trend method. In the simulation based on the mouse data, the NBPSeq:genewise method outperforms all other methods for finding the first 25% of truly DE genes (i.e., in the plot region where TPR ≤ 0.25), but it is eventually outperformed by QuasiSeq:QLSpline and edgeR:tagwise-trend if a greater percentage of truly DE genes need to be detected. Similar trend is observed in simulations based on the zebrafish and fruit fly datasets. This indicates the NBPSeq:genewise method can have advantage for detecting DE genes with small fold changes. There is less separation between QuasiSeq:QLSpline and edgeR:tagwise-trend methods when the DE fold changes were specified to be 1.5. Again, the performance of all methods assuming a dispersion model (i.e., all methods except NBPSeq:genewise) improves significantly when the residual dispersion variation is halved.

#### FDR and Type-I Error

In practice, the Benjemini-Hochberg method [29] is commonly used to control the FDR of DE tests. In Table 4, we compare the *actual* FDR of the different DE tests based on the simulation results when the *nominal* FDR is set to 10% using the Benjemini-Hochberg method. The results are based on the datasets simulated to mimic the human dataset, where we vary the percentage of DE genes (10% and 20%) and we vary *σ* from estimated value ($\sigma =\tilde{\sigma }$), to half the estimated value ($\sigma =0.5\tilde{\sigma }$), and then to 0. We consider three ways to specify fold changes (FC) for DE genes: estimated from data, FC = 1.2 and FC = 1.5. The QuasiSeq:QLSpline and NBPSeq:genewise methods have good controls on FDR in all cases, and are conservative in some cases. The edgeR:tagwise-trend method has good FDR control when the percentage of DE genes is high (20%), but underestimates FDR in several cases when the percentage of DE genes is low (10%). For the NBPSeq:NBQ and edgeR:trended methods, the FDR control improves as the residual dispersion variation decreases and as the percentage of truly DE genes increases. The edgeR:common method does not have good control of FDR in almost all scenarios.

**Table 4:** Actual FDR for a nominal FDR of 0.1.

			**Actual FDR for 10% Nominal FDR**					
Fold Change	*σ*	%DE	Genewise	QLSpline	Tagwise-Trend	NBQ	Trended	Common
	$\tilde{\sigma }$	10%	9.72%	7.84%	12.5%	28.3%	29.3%	39.6%
		20%	7.90%	6.57%	6.76%	17.3%	18.2%	25.3%
Estimated from data	$0.5\tilde{\sigma }$	10%	11.8%	10.5%	14.2%	13.3%	14.4%	38.3%
		20%	10.7%	8.03%	11.5%	13.4%	14.1%	23.9%
	0	10%	11.2%	10.2%	16.2%	10.6%	11.9%	26.6%
		20%	7.19%	5.61%	9.88%	6.86%	7.46%	17.4%
	$\tilde{\sigma }$	10%	9.72%	10.5%	10.4%	35.9%	37.3%	54.8%
		20%	5.23%	7.05%	9.48%	18.6%	19.3%	28.6%
FC = 1.2	$0.5\tilde{\sigma }$	10%	11.3%	10.0%	14.6%	16.0%	17.8%	27.9%
		20%	7.87%	8.79%	9.54%	10.1%	10.8%	18.7%
	0	10%	10.7%	9.47%	13.1%	9.09%	9.72%	22.3%
		20%	8.72%	9.83%	12.5%	9.68%	10.2%	16.0%
	$\tilde{\sigma }$	10%	9.92%	10.8%	12.9%	19.0%	19.9%	23.4%
		20%	7.26%	7.17%	7.06%	11.6%	12.2%	15.1%
FC = 1.5	$0.5\tilde{\sigma }$	10%	7.13%	7.25%	9.87%	12.1%	13.2%	17.5%
		20%	6.11%	5.78%	7.40%	9.13%	9.38%	13.5%
	0	10%	9.71%	7.59%	12.8%	8.32%	9.14%	18.7%
		20%	7.53%	10.4%	9.70%	7.63%	7.63%	11.6%

We consider three settings for DE fold changes (estimated from data, fixed at 1.2 and fixed at 1.5), three levels of *σ* (the estimated value $\sigma =\tilde{\sigma }$, half the estimated value $\sigma =0.5\tilde{\sigma }$, and no variation *σ* = 0) and two levels of percentage of DE genes (10% and 20%).

**Fig 5:**
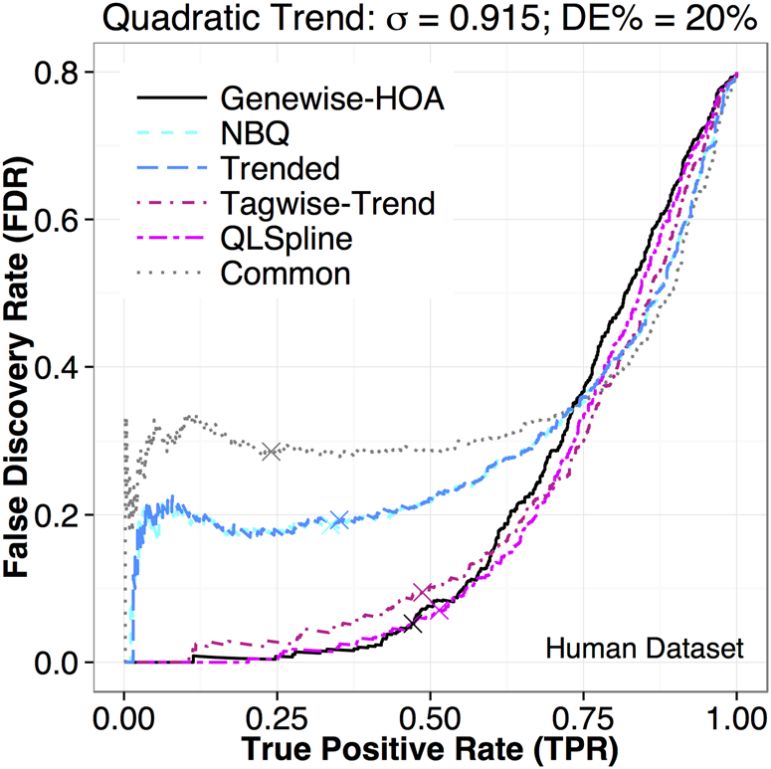
True Positive Rate (TPR) vs. False Discovery Rate (FDR) plots for the six DE test methods performed on RNA-Seq dataset simulated to mimic the human dataset. On each curve, we marked the position corresponding to a reported FDR of 10% with a cross. The fold changes of DE genes are fixed at 1.2 (half of the DE genes are over-expressed and the other half are under-expressed). Other simulation settings are identical to those for the upper row of Fig. 2.

Fig. 5 shows what will happen if one uses the reported FDR to identify DE genes. We uses one of the simulated human data as an example (the fold change is specified to be 1.2 for the designated 20% DE genes, and $\sigma =\tilde{\sigma }$), since the tests are well separated here. For methods that do not correctly control FDR, such as NBPSeq:NBQ and edgeR:trended, if one identifies DE genes according to a cutoff on reported FDR (e.g., 10%), more genes will be detected as DE (than if one were able to use the actual FDR) at the cost of underestimated FDR.

**Fig 6:**
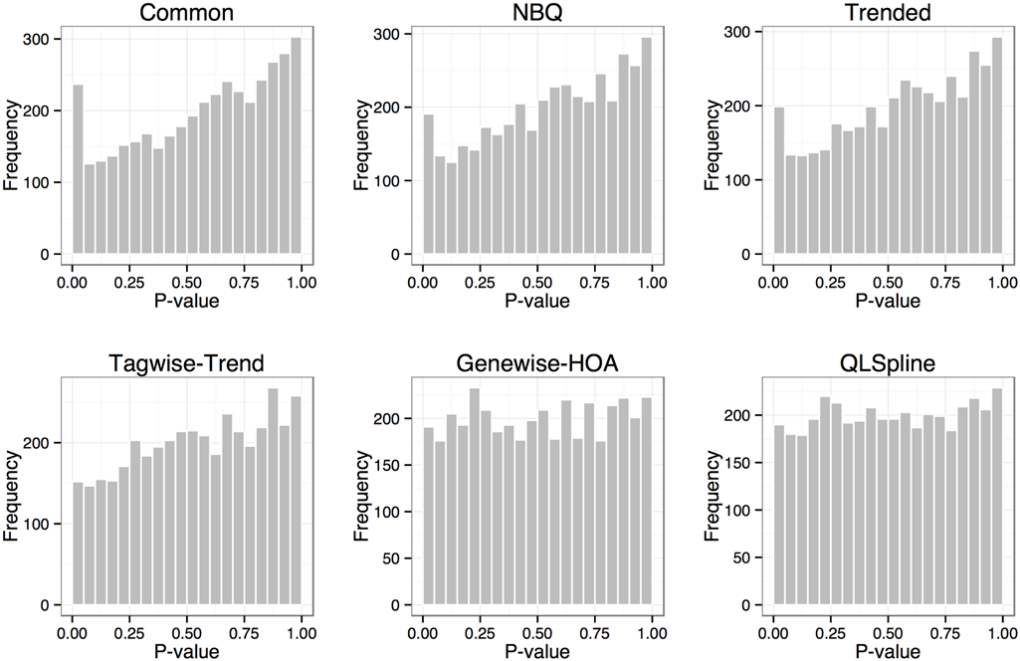
Histograms of *p*-values for the non-DE genes from the six DE test methods. The simulation dataset is based on the human dataset with *σ* specified as the estimated value $\sigma =\tilde{\sigma }$. Out of a total of 5,000 genes, 80% are non-DE.

The FDR control is closely related to the test *p*-values. Fig. 6 shows the histograms of *p*-values computed for the non-DE genes in one of the datasets used for the FDR comparison above (fold change estimated from data, 20% DE and $\sigma =\tilde{\sigma }$). The histograms from the NBPSeq:genewise and QuasiSeq:QLSpline methods are replacedclosermore close to uniform. For the edgeR:common, NBPSeq:NBQ and edgeR:trended methods, the histograms are asymmetric v-shaped: there is an overabundance of small *p*-values as compared to a uniform distribution, but the histograms also indicate that these tests are conservative for many genes. Similar patterns have been observed for other dispersion-modeling methods by Lund *et al.* in [18]. The edgeR:tagwise-trend method produces conservative *p*-values.

**Fig 7:**
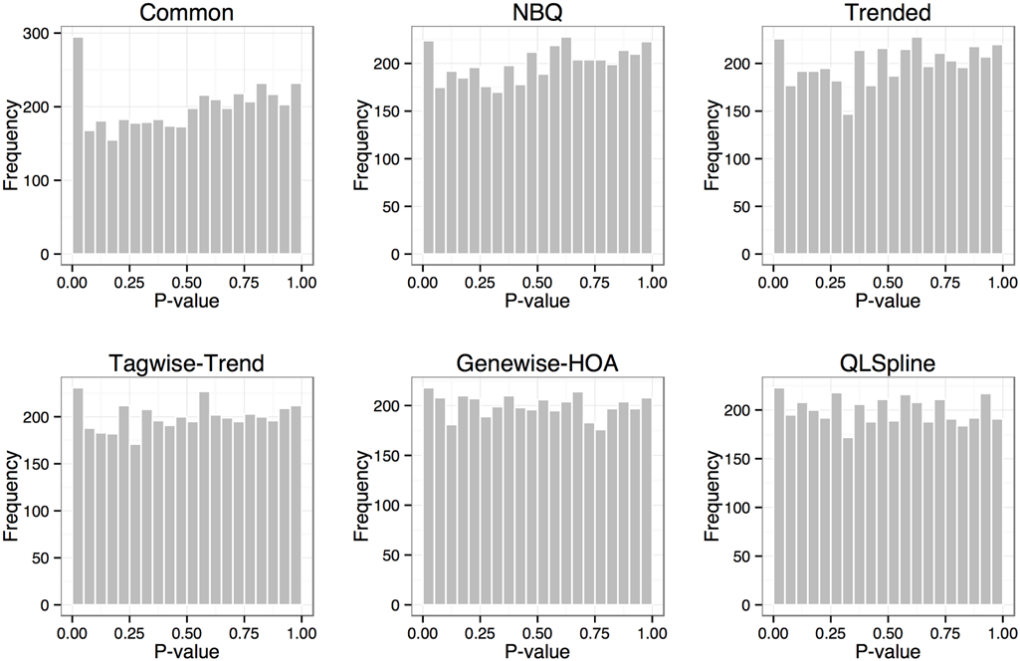
Histograms of *p*-values for the non-DE genes from the six DE test methods. The simulation dataset is based on the human dataset with *σ* specified as half the estimated value $\sigma =0.5\tilde{\sigma }$. Out of a total of 5,000 genes, 80% are non-DE.

Fig. 7 shows similar histogram comparisons when *σ* was reduced to half the estimated value ($0.5\tilde{\sigma }$), while fold change and DE percentage remained the same. The null *p*-value histograms from the NBPSeq:NBQ and edgeR:trended methods have improved and are closer to the uniform distribution. The edgeR:tagwise-trend method produces a slight overabundance of small *p*-values. The edgeR:common method is still unsatisfactory.

## Conclusion and Discussion

We quantified the residual dispersion variation in five real RNA-Seq datasets. Using simulations, we compared the performance—in terms of power and FDR/Type-I error control—of six representative DE test methods based different dispersion models. We demonstrated that the level of residual dispersion variation is a crucial factor in determining the performance of DE tests. When the residual dispersion variation is as high as we estimated from the five real datasets, methods such as NBPSeq:NBQ and edgeR:trended, which ignore possible residual dispersion variation, fail to control Type-I errors and give suboptimal power. The QuasiSeq:QLSpline and edgeR:tagwise-trend methods have similar size-corrected power, but the edgeR:tagwise-trend method underestimates FDR when the percentage of DE genes is low or when the fold changes of the DE genes is low. QuasiSeq:QLSpline and edgeR:tagwise-trend both account for individual dispersion variation. QuasiSeq:QLSpline also makes degrees-of-freedom adjustment to address the uncertainty in estimated NB dispersions. Based on these observations, we recommend incorporating individual variation and using degrees-of-freedom adjustment to improve robustness and Type-I error control for DE test methods that use a dispersion model.

The NBPSeq:genewise method does not rely on a dispersion model, and it uses an HOA technique to improve small-sample performance of the likelihood ratio test. The NBPSeq:genewise method has good Type-I error and FDR control in all simulations. The power of the NBPSeq:genewise method is comparable to that of the QuasiSeq:QLSpline and edgeR:tagwise-trend methods when the level of residual dispersion variation is high. This indicates that when the level of dispersion variation is high, the power saving available through dispersion modeling is limited.

Reducing the level of dispersion variation boosts the performance of DE tests that use a dispersion model. One may attempt to improve the dispersion model by considering different functional forms of the trend and/or including additional predictors. We plan to explore such possibilities in our future research. It is not well understood what factors contribute to the count and dispersion variation in an RNA-Seq experiment: possible factors to consider include transcript length, GC-content, and so on.

**Fig 8:**
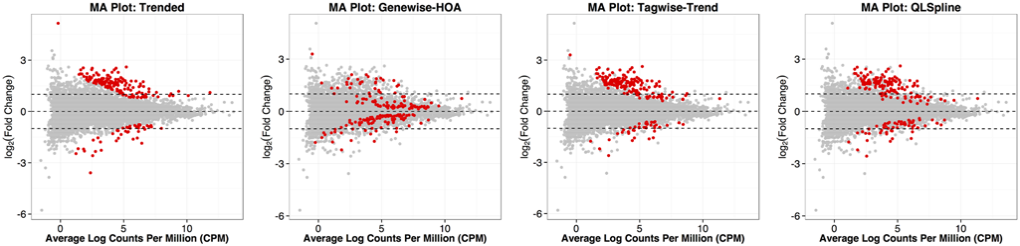
MA plots for the edgeR:trended, NBPSeq:genewise, edgeR:tagwise-trend and QuasiSeq:QLSpline methods performed on the mouse dataset. Predictive log fold changes (posterior Bayesian estimators of the true log fold changes, the “M” values) are shown on the *y*-axis. Averages of log counts per million (CPM) are shown on the *x*-axis (the “A” values). The M- and A- values are calculated using edgeR. The highlighted points correspond to the top 200 DE genes identified by each of the DE test methods.

One notable difference between the NBPSeq:genewise method and a dispersion-modeling method is that the former detects more DE genes with small fold changes, while a method using a dispersion model tends to detect DE genes with large fold changes. This phenomenon agrees with what we observed in the power simulation when the DE fold change was fixed to be low, 1.2. Fig. 8 illustrates this point using MA plots. This is because current dispersion models often assume the dispersion is the same for genes with similar mean levels (those genes having the same *x*-values). Under such assumptions, large fold changes tend to correspond to more significant test results. The behaviors of the edgeR:tagwise-trend and the QuasiSeq:QLSpline methods are intermediate between the NBPSeq:genewise method and a dispersion-modeling method such as the edgeR:trended model.

For the six methods we compared, edgeR:common, edgeR:trended, edgeR:tagwise-trended use likelihood ratio test. NBPSeq:genewise and NBPSeq:NBQ use HOA-adjusted likelihood ratio test. From our past studies, we know that HOA adjustment mainly corrects for Type-I error and does not significantly change the power when compared to the unadjusted likelihood ratio test. So the differences between the these five methods in the power comparison are mainly attributable to how they handle the dispersion estimation, especially with respect to the two factors highlighted in Table 3: 1) whether they consider a trend *f* in log dispersion, and 2) whether they consider possible additional individual variation *ε*_*i*_. The HOA adjustment in NBPSeq may have contributed to the different Type-I error performances. QuasiSeq:QLSpine uses a different test for DE and differs from the above five methods in more aspects. Regarding the dispersion estimation, it considers the general trend *f* in the dispersion, considers additional individual variation, and uses some degree-of-freedom adjustment. We believe all three aspects contributed to its performance.

We used a 𝒩(0,*σ* ^2^) distribution to model the residual dispersion variation *ε*_*i*_ (see Equation (2)). We believe this is a reasonable starting point. The authors in [23] made a similar assumption and used simple diagnostic plots to show the normality assumption was reasonable. To rigorously test this assumption, however, is challenging due to the small sample size. It might be more useful to consider alternative model assumptions on *ε*, compare results and investigate sensitivity to model assumptions. In future, we will also consider the possibility that *σ* may vary with some other variables, such as the mean level. However, the general conclusion that the performance of the DE tests depends on the level of the residual dispersion variation should remain valid.

## Methods

### Description of RNA-Seq Datasets

Experiment information for all species and the raw/processed data are available at the Gene Expression Omnibus (GEO) of the National Center for Biotechnology Information (NCBI). Table 5 gives a brief summary of the datasets analyzed in this paper, including the dataset names in the SeqDisp R package we develop (see the Software Information section), the SRA accessions that provides all the metadata describing a particular study (see the NCBI website for different accession types), and published references. In the Supporting Information S1 File, see “Access to the Datasets” section and Table A for more details.

**Table 5:** Summary of RNA-Seq datasets analyzed in this article.

**Organism**	**Name in SeqDisp**	**SRA Accession**	**References**
*Homo Sapiens*	human30/human5	SRP031476	[30]
*Mus Musculus*	mouse	SRP022850	[31]
*Danio Rerio*	zebrafish	SRP017511	[32]
*Arabidopsis Thaliana*	arabidopsis	SRP013873	[33]
*Drosophila Melanogaster*	fruit.fly	SRP001537	[34, 35]

#### Human RNA-Seq Data

The *Homo sapiens* (human) RNA-Seq experiment was discussed in [30]. In this study, researchers compared the gene expression profiles for human cell line MCF7 cells (from American Type Cell Culture) under treatment (10 nM 17*β*-estradiol (E2)) versus control. Information for this experiment, the raw and processed data are available at NCBI GEO under accession number GSE51403.

Liu *et al.* [30] focused more on the technical side of RNA-Seq experiments by investigating the trade-offs between sequencing depth (where a higher depth generates more informational reads) and the number of biological replicates. Seven biological replicates of both control and E2-treated MCF7 cells were sequenced, and the RNA-Seq reads in each sample were down-sampled to generate datasets of different depths (a total of seven depths from 2.5M to 30M). We include datasets from two sequencing depths (5M and 30M) in our R package, but mainly focus on the dataset with 30M sequencing depth for analyses. See [30] and NCBI GSE51403 for detailed descriptions of the dataset.

#### Mouse RNA-Seq Data

The *Mus musculus* (mouse) RNA-Seq experiment was discussed in [31]. This experiment used RNA-Seq to study the impact of competent versus abnormal human embryos on endometrial receptivity genes in the uteri of 25-day wild-type C57BL/6 mice. Information for this experiment and the raw data are available at NCBI GEO under accession number GSE47019. The raw data are downloaded from NCBI Sequence Read Archive (SRA), and processed using the pipeline described in [27].

We summarize the samples of “Control Salker”, “Developmentally competent embryo conditioned media Salker” (abbreviated as DCECM) and “Arrested embryo conditioned media Salker” (abbreviated as AECM) into the mouse dataset in the SeqDisp R package. We only consider the control and DCECM groups in the analyses.

#### Zebrafish RNA-Seq Data

The *Danio rerio* (zebrafish) RNA-Seq experiment was discussed in [32], and information for this experiment and the raw data are available at NCBI GEO under accession number GSE42846. This study compared gene expression profiles of zebrofish embryos infected with Staphylococcus epidermidis versus control. Four biological replicates are prepared for the control group (Non-injected 5 DPI) and for the treatment group (S. epi mcherry O-47 5 DPI).

#### Arabidopsis RNA-Seq Data

The *Arabidopsis thaliana* (Arabidopsis) RNA-Seq experiment was discussed in [33], and information for this experiment and the raw data are available at NCBI GEO under accession number GSE38879. This study analyzed 7 days old seedlings from two lines of Arabidopsis (rve8-1 RVE8::RVE8:GR and rve8-1) treated with dexamethasone or mock. The overall design includes transgenic line rve8-1 RVE8::RVE8:GR and rve8-1 treated with DEX or mock with three biological replicates each, for a total of 12 samples. Our analyses only focus on the RVE8:GR_mock control group, and the RVE8:GR_DEX treatment group.

#### Fruit Fly RNA-Seq Data

The *Drosophila melanogaster* (fruit fly) RNA-Seq experiment was discussed in [34], and information for this experiment and the raw data are available at NCBI GEO under accession numbers GSM461176 to GSM461181. The experiment compared gene expression profiles of fruit fly S2-DRSC cells (FlyBase cell line) depleted of mRNAs encoding RNA biding proteins versus control. The dataset fruit.fly in our SeqDisp package is directly obtained from the pasilla Bioconductor package [35], which provides per-exon and per-gene read counts computed for selected genes in [34]. It can also be accessed from data(pasillaGenes) once pasilla is loaded. The dataset contains three and four biological replicates of the knockdown and the untreated control, respectively. See the pasilla package vignette for more information.

### Quantifying the Level of Residual Dispersion Variation

#### Estimating *σ* ^2^

In the RNA-Seq context, we use *Y*_*ij*_ to denote the read count for gene *i* in sample *j*, where *i* = 1,⋯,*m* and *j* = 1,⋯,*n*. We model a single read count as negative binomial with mean *μ*_*ij*_ and dispersion *ϕ*_*ij*_:

$$\begin{array}{c} Y_{ij}∼\mathrm{NB}\left(\mu_{ij},\phi_{ij}\right), \end{array}$$

and assume a log-linear model for *μ*_*ij*_, i.e., $\log(\mu_{ij})=\text{offset}+X_{j}^{\prime }\beta_{i}$ (see also Equation (3)). We further assume a parametric distribution as the prior distribution for the dispersion parameter *ϕ*_*ij*_:

$$\begin{array}{c} \log\left(\phi_{ij}\right)=\log\left(\phi_{ij}^{0}\right)+\epsilon_{i}, \end{array}$$

where *ε*_*i*_ ∼ 𝒩(0,*σ* ^2^). The prior mean, $\log(\phi_{ij}^{0})$, is preliminarily estimated according to a dispersion model (e.g., NBQ or a smooth fit like NBS) and is treated as known. Our goal is to estimate *σ* ^2^.

Let *θ*_*ij*_ = log(*ϕ*_*ij*_) and $\theta_{ij}^{0}=\log(\phi_{ij}^{0})$, so that $\theta_{ij}=\theta_{ij}^{0}+\varepsilon_{i}$. Across all *m* genes, we assume that *ε*_*i*_’s are independent, and denote the prior distribution of *ε*_*i*_ by *π*(*ε*_*i*_∣*σ* ^2^). The joint likelihood function of the unknown parameters (*σ* ^2^,*β*) is

$$\begin{array}{c} L\left(\sigma^{2},\beta \right)=\prod_{i=1}^{m}\int L_{i}\left(\beta_{i}|\epsilon_{i}\right)\pi \left(\epsilon_{i}|\sigma^{2}\right)d\epsilon_{i}, \end{array}$$ (8)

where *L*_*i*_(*β*_*i*_∣*ε*_*i*_) is the likelihood of *β*_*i*_ from gene *i* for a given *ε*_*i*_:

$$\begin{array}{c} L_{i}\left(\beta_{i}|\epsilon_{i}\right)=\prod_{j=1}^{n}\mathrm{Pr}\left(y_{ij}|\theta_{ij}=\theta_{ij}^{0}+\epsilon_{i},\beta_{i}\left(\theta_{i}\right)\right). \end{array}$$

We want to estimate *σ* ^2^ by maximizing the profile likelihood of *σ* ^2^:

$$\begin{array}{c} L_{p}\left(\sigma^{2}\right)=\max_{\beta }L\left(\sigma^{2},\beta \right). \end{array}$$ (9)

It is difficult to maximize *β*_*i*_ with respect to an integrated likelihood. We instead consider

$$\begin{array}{c} L_{p}\left(\sigma^{2}\right)\approx \prod_{i=1}^{m}\int L_{i}\left({\hat{\beta }}_{i}\left(\epsilon_{i}\right)|\epsilon_{i}\right)\pi \left(\epsilon_{i}|\sigma^{2}\right)d\epsilon_{i}, \end{array}$$ (10)

where ${\hat{\beta }}_{i}(\varepsilon_{i})$ is the MLE of *β*_*i*_ for fixed *ε*_*i*_ (and thus fixed *ϕ*_*ij*_). ${\hat{\beta }}_{i}(\varepsilon_{i})$ can be obtained by the standard iteratively reweighted least squares algorithm [36].

Let $l_{i}(\varepsilon_{i})=\log\left(L_{i}({\hat{\beta }}_{i}(\varepsilon_{i})|\varepsilon_{i})\right)$ and *π*(*ε*_*i*_∣*σ* ^2^) be the normal density. Equation (10) can be rewritten as

$$\begin{array}{c} L_{p}\left(\sigma^{2}\right)\approx \prod_{i=1}^{m}\frac{1}{\sqrt{2\pi \sigma^{2}}}\int \exp\left(l_{i}\left(\epsilon_{i}\right)-\frac{\epsilon_{i}^{2}}{2\sigma^{2}}\right)d\epsilon_{i}. \end{array}$$ (11)

The dependence on *y*_*ij*_ is implicit through *l*_*i*_(*ε*_*i*_) in Equation (11). We approximate the integral in Equation (11) using the Laplace’s method [37]. Let $\varepsilon_{i}^{*}$ maximize

$$\begin{array}{c} g_{i}\left(\epsilon_{i}\right)=l_{i}\left(\epsilon_{i}\right)-\frac{\epsilon_{i}^{2}}{2\sigma^{2}}, \end{array}$$

so that

$$\begin{array}{c} g_{i}^{'}\left(\epsilon_{i}^{*}\right)=l_{i}^{'}\left(\epsilon_{i}^{*}\right)-\frac{\epsilon_{i}^{*}}{\sigma^{2}}=0. \end{array}$$

Then $\frac{1}{\sqrt{2\pi \sigma^{2}}}\int \exp\left(l_{i}(\varepsilon_{i})-\frac{\varepsilon_{i}^{2}}{2\sigma^{2}}\right)d\varepsilon_{i}$ in Equation (11) can be approximated by

$$\begin{array}{c} \frac{1}{\sqrt{-\sigma^{2}g_{i}^{''}\left(\epsilon_{i}^{*}\right)}}\exp\left(g_{i}\left(\epsilon_{i}^{*}\right)\right)=\frac{1}{\sqrt{\sigma^{2}\left(-l_{i}^{''}\left(\epsilon_{i}^{*}\right)+1/\sigma^{2}\right)}}\exp\left(l_{i}\left(\epsilon_{i}^{*}\right)-\frac{{\epsilon_{i}^{*}}^{2}}{2\sigma^{2}}\right). \end{array}$$

#### Evaluation of $\hat{\sigma }$

**Fig 9:**
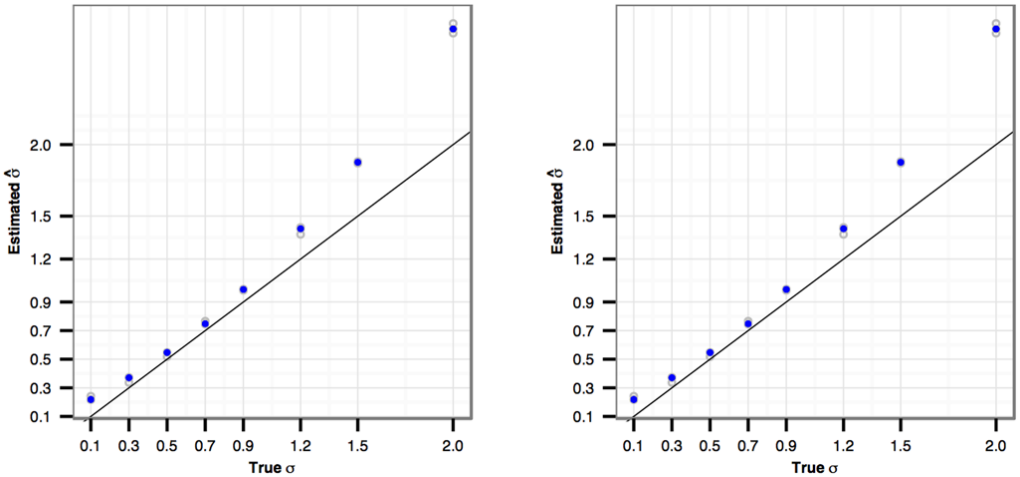
Estimation accuracy of $\hat{\sigma }$. In the simulation, the dispersion is simulated according to an NB2 (left panel) or an NBQ (right panel) trend with added individual variation *ε*_*i*_ ∼ 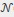
(0,*σ* ^2^). The *x*-axis is the true *σ* value and the *y*-axis is the estimated $\hat{\sigma }$. For each true *σ* value, the simulation is repeated three times. The blue dots correspond to the median $\hat{\sigma }$ values.

To evaluate the estimation accuracy for *σ*, we perform a set of simulations using the human RNA-Seq dataset as the “template” in order to preserve observed relationships between the dispersions and gene-specific mean counts. We simulate 5,000 genes with a single group of seven replicates: the mean structure *μ* is randomly generated according to a log-normal distribution with mean 8.5 and standard deviation 1.5 (both on the log scale and the values are chosen to mimic the real dataset); the trend of the dispersion is estimated from the real dataset according to an NB2 or an NBQ model; individual residual variation *ε*_*i*_ is simulated according to 𝒩(0,*σ* ^2^) and added to the trend. We compare $\hat{\sigma }$ with true *σ* specified at eight levels that are within a reasonable range for typical RNA-Seq data: 0.1, 0.3, 0.5, 0.7, 0.9, 1.2, 1.5 and 2.0. At each level of *σ* we repeated the simulation three times using different random number seeds for generating *ε*_*i*_ ∼ 𝒩(0,*σ* ^2^). Fig. 9 shows the simulation results. We highlight the median value (out of three repetitions) in solid blue point at each *σ* level, and ideally these points should follow the *y* = *x* reference line. We see that there is some bias in the estimation. The bias will increase for smaller sample sizes. We see that $\hat{\sigma }$ is more accurate for *σ* values between 0.3 and 0.9 and less so for *σ* values outside this range. The results (not shown) are similar when we use the NBP (log-linear) and NBS (smooth function) models to capture the general trend in the dispersion.

#### Calibration

**Fig 10:**
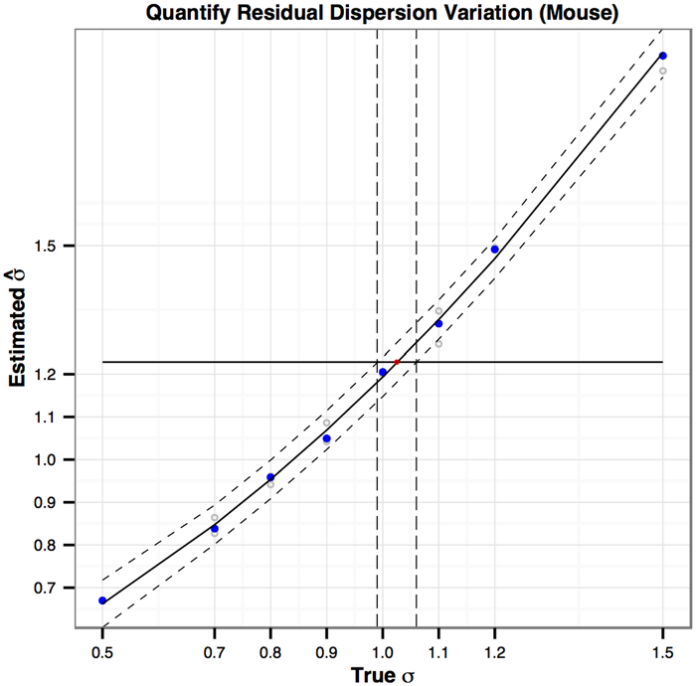
The calibration plot for estimating residual dispersion variation *σ* for the mouse dataset. The *x*-axis is the *σ* value used to generate the data. The *y*-axis is the estimated $\hat{\sigma }$. The horizontal line correspond to the $\hat{\sigma }$ estimated from the mouse dataset.

As discussed in the “Results/Power-Robustness Evaluations/Simulation Setup” subsection, when simulating the RNA-Seq datasets, we want to choose a *σ* that matches the level of residual dispersion variation in real data. We want to correct for potential bias in the estimator $\hat{\sigma }$. We also need to account for the discrepancy between *π*_*ij*_ (used when simulating the data) and ${\hat{\pi }}_{ij}$ (used when fitting the dispersion model). This is achieved by a calibration approach [38]. The calibrated $\tilde{\sigma }$’s are essentially obtained from a calibration plot. Fig. 10 shows the calibration plot for the mouse dataset (subsetted to 5,000 genes). We choose the *σ* value at eight levels: 0.5, 0.7, 0.8, 0.9, 1.0. 1.1, 1.2 and 1.5, and simulate the dispersion *ϕ*_*ij*_ according to

$$\begin{array}{c} \log\left(\phi_{ij}\right)=\log\left(\phi_{ij}^{\mathrm{NBQ}}\right)+\epsilon_{i}=\alpha_{0}+\alpha_{1}\log\left(\pi_{ij}\right)+\alpha_{2}{\left[\log\left(\pi_{ij}\right)\right]}^{2}+\epsilon_{i}, \end{array}$$

where *ε*_*i*_ ∼ *N*(0,*σ* ^2^) is the residual variation on top of an NBQ dispersion model with the parameters *α*_*i*_,*i* = 0,1,2, estimated from the mouse dataset. At each level of *σ*, we simulate three datasets and obtain three $\hat{\sigma }$’s. We then fit a quadratic curve to the eight median $\hat{\sigma }$ values as a function of *σ*, with a 95% prediction interval superimposed in dashed curves. The $\hat{\sigma }$ estimated from the mouse dataset is also calculated, and the value is shown as a horizontal solid line. The intersection of the fitted quadratic curve and the horizontal line (the solid red point) has its *x* coordinate being the calibrated $\tilde{\sigma }$. Similarly, the intersections between the upper/lower bound of the 95% prediction interval with the horizontal line determine the associated 95% calibration interval (CI) for the calibrated $\tilde{\sigma }$. We only include the calibration plot for the mouse dataset as an illustration. Table 6 summarizes the calibrated $\tilde{\sigma }$ with 95% CI for each of the five real datasets.

**Table 6:** Calibrated $\tilde{\sigma }$ values for the five real datasets.

**Dataset**	**#samples**	**Calibrated $\tilde{\sigma }$**	**95% CI**
Human30	(7, 7)	0.915	(0.90, 0.93)
Mouse	(3, 3)	1.025	(0.99, 1.06)
Zebrafish	(4, 4)	0.975	(0.94, 1.02)
Arabidopsis	(3, 3)	0.855	(0.80, 0.92)
Fruit fly	(4, 3)	0.890	(0.86, 0.93)

We also report the 95% calibration interval (CI) for each point estimate.

### Software Information

The proposed approach of estimating the level of residual dispersion variation *σ* is implemented as an R package named SeqDisp (released at https://github.com/gu-mi/SeqDisp, under GPL-2 License). The package also provides graphical functionality to generate diagnostic plots for comparing different dispersion methods. All datasets (raw read count tables) analyzed in this article are included in the package. The R codes for reproducing all results in this article are available at the first author’s github page.

## Supporting Information

S1 File Supplementary Information on Datasets, Plots and Discussions. Access information to the datasets analyzed in this article (Table A), the mean-dispersion plots (Figs. A–D), and discussion of the relationship between $\hat{\sigma }$ and ${\hat{d}}_{0}$ (Fig. E) are provided in the Supporting Information S1 File.
(PDF)
