# Supplementary material for: The Level of Residual Dispersion Variation and the Power of Differential Expression Tests for RNA-Seq Data
Source: PLoS One. 2015 Apr 7;10(4):e0120117. doi: 10.1371/journal.pone.0120117 (PMC4388866; doi:10.1371/journal.pone.0120117)
Supplement: S1 File — Access information to the datasets analyzed in this article (Table A), the mean-dispersion plots (Figs. A–D), and discussion of the relationship between σ^ and d^0 (Fig. E) are provided in the Supporting Information S1 File. (PDF) [file pone.0120117.s001.pdf]

Supporting Information for The Level of Residual Dispersion Variation and the Power of Differential Expression Tests for RNA-Seq Data

Gu Mi[1,*], Yanming Di[1,2]
1 Department of Statistics, Oregon State University, Corvallis, Oregon, United States of America
2 Molecular and Cellular Biology Program, Oregon State University, Corvallis, Oregon, United States of America
∗ **E-mail: neo.migu@gmail.com**

Access to the Datasets

Information for all the datasets we analyzed in this article can be accessed from the NCBI website, using the GEO DataSets Advanced Search Builder. To obtain all the relevant information for an interested species (e.g., experiment descriptions, raw/processed data files, protocols and publications, etc.), we search in the "Organism" box and restrict the scope of "expression profiling by high throughput sequencing" in the "Filter" box.

The following datasets in the `SeqDisp` package contain read counts for all the samples in the original experiments: `human5`, `human30`, `mouse`, `zebrafish` and `arabidopsis`. For the `fruit.fly` dataset, as indicated in the `pasilla` package vignette, we only include read counts for seven samples. See Table A for the accession numbers and sequencing platforms for each of the datasets.

Table A. Additional information for RNA-Seq datasets analyzed in this article.

Organism	Accession Number	Platform
Homo Sapiens	GSM1244809 – GSM1244816	Illumina HiSeq 2000
Mus Musculus	GSM1143032 – GSM1143040	Illumina HiSeq 2000
Danio Rerio	GSM1051294 – GSM1051301	Illumina HiSeq 2000
Arabidopsis Thaliana	GSM951349 – GSM951360	Illumina HiSeq 2000
Drosophila Melanogaster	GSM461176 – GSM461181	Illumina Genome Analyzer II

Supplementary Figures for Mean-Dispersion Plots

In the main text, we showed the mean-dispersion plot in Fig. 1 for the human dataset (with sequencing depth of 30 million). Here we provide such mean-dispersion plots for the mouse (Fig. A), zebrafish (Fig. B), Arabidopsis (Fig. C) and fruit fly (Fig. D) datasets. The left panels are for the control groups, and the right panels are for the treatment groups.

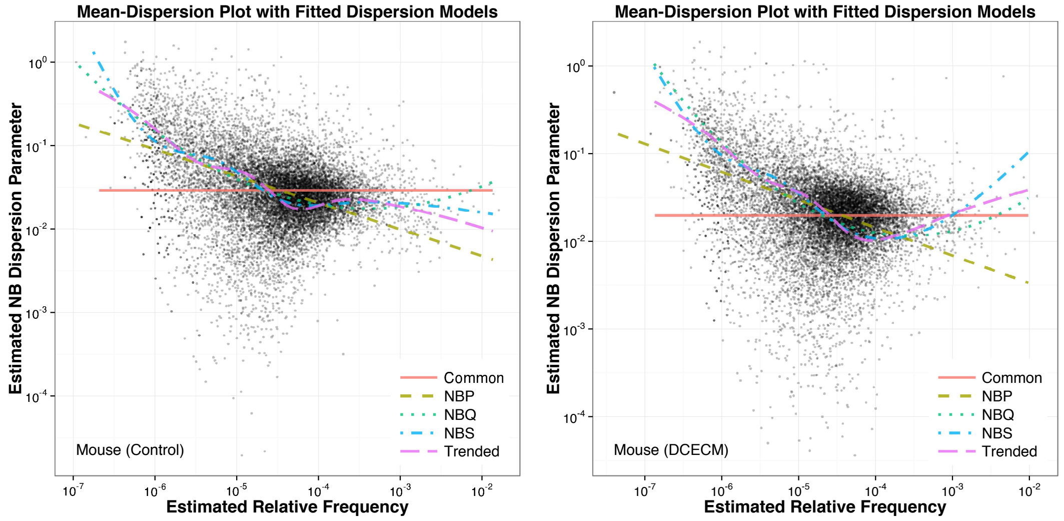

Figure A. Mean-dispersion plot of the mouse RNA-Seq dataset. The control (DCECM) group with three biological replicates is shown on the left (right) panel.

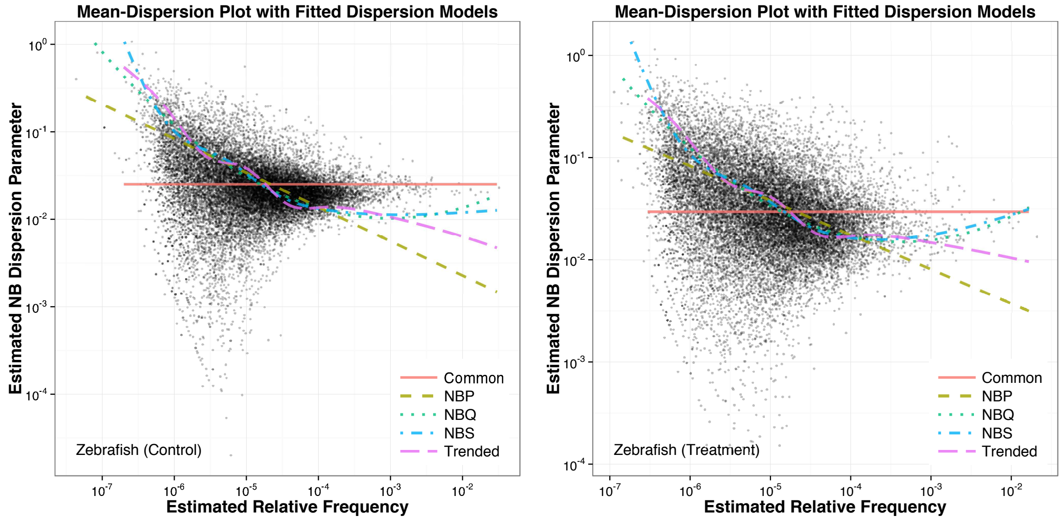

Figure B. Mean-dispersion plot of the zebrafish RNA-Seq dataset. The control (treatment) group with four biological replicates is shown on the left (right) panel.

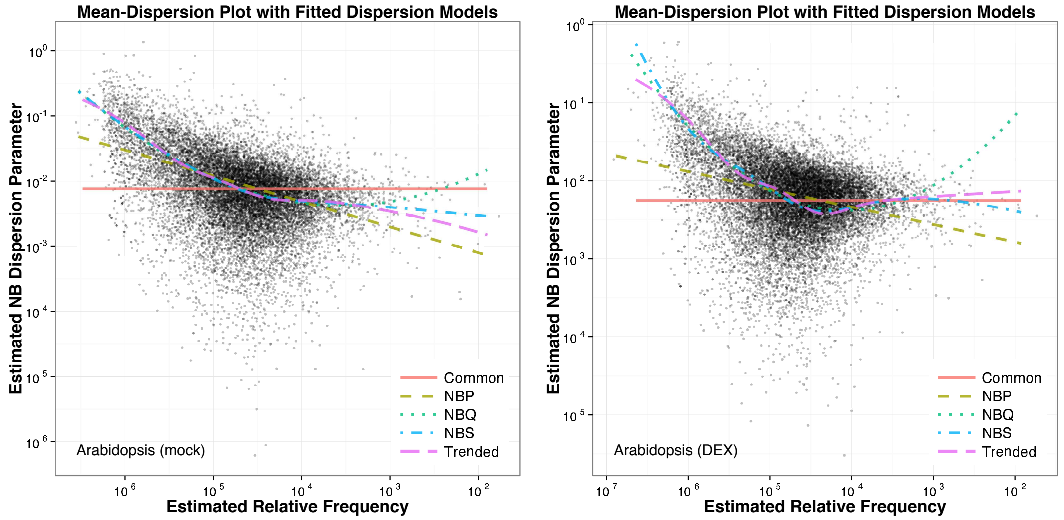

Figure C. Mean-dispersion plot of the Arabidopsis RNA-Seq dataset. The mock (DEX) group with three biological replicates is shown on the left (right) panel.

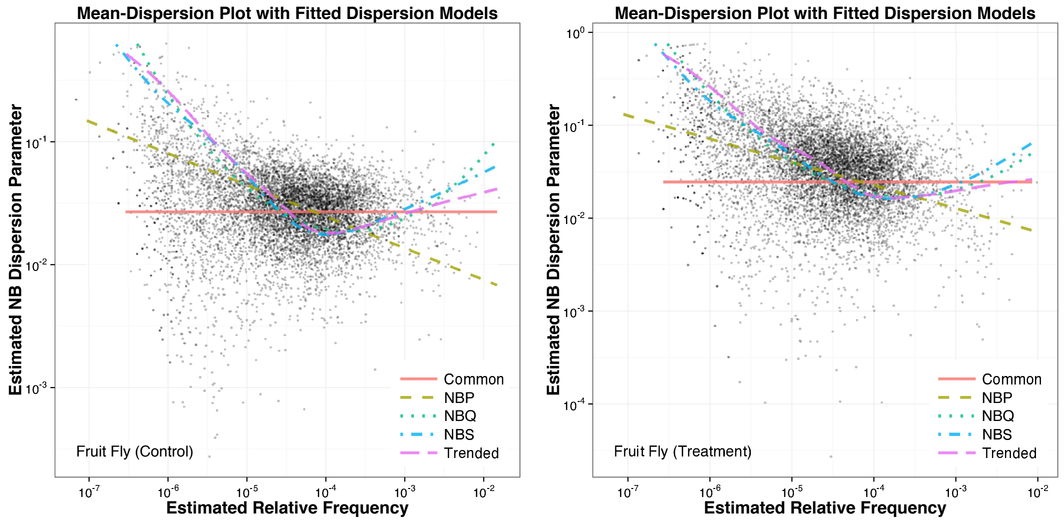

Figure D. Mean-dispersion plot of the fruit fly RNA-Seq dataset. The untreated control (knockdown treatment) group with four (three) biological replicates is shown on the left (right) panel.

Supplementary Figure for Relationship Between $\hat{\sigma}$ and $\hat{d}_0$

In the main text, we discussed the relationship between our proposed measure of residual dispersion variation σ, and the prior degrees of freedom d_0 in the empirical Bayes framework. Here we provide the simulation result in Fig. E which illustrates this inversely proportional relationship (based on the mouse dataset). At each of the eight true σ values of $0.5, 0.7, 0.8, 0.9, 1.0, 1.1, 1.2$ and 1.5, we calculated the corresponding values of $\hat{\sigma}$ and $\hat{d}_0$, and plotted the pairs in Fig. E. The $\hat{d}_0$ was computed using the `estimateDisp` function in the `edgeR` Bioconductor package (version 3.6.2).

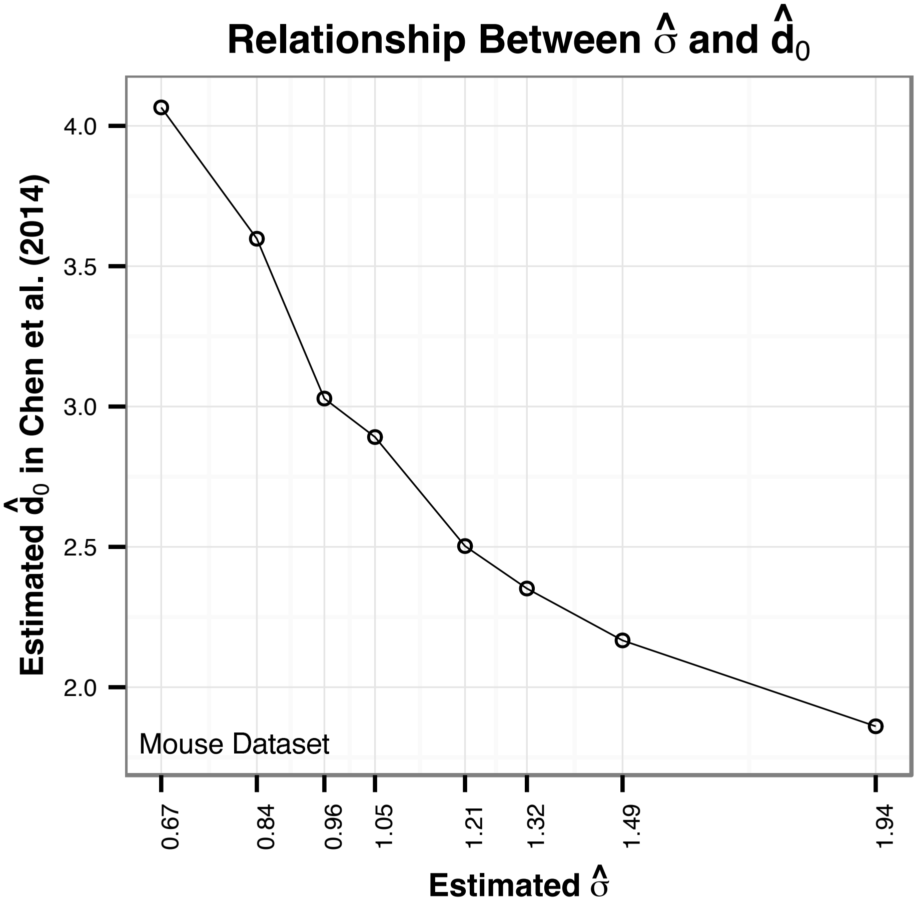

Figure E. Relationship between $\hat{\sigma}$ (using the approach discussed in the main text) and $\hat{d}_0$ (prior degrees of freedom) discussed in Chen *et al.* (2014). Results are based on the mouse dataset with identical simulation setup as in Fig. 8 of the main text.
